# Biological signatures in the Alzheimer’s continuum discriminate between diagnosis-related and -unrelated associations to ATN categories

**DOI:** 10.1093/braincomms/fcaf078

**Published:** 2025-02-21

**Authors:** Vilma Alanko, Sára Mravinacová, Anette Hall, Göran Hagman, Rosaleena Mohanty, Eric Westman, Peter Nilsson, Miia Kivipelto, Anna Månberg, Anna Matton

**Affiliations:** Division of Clinical Geriatrics, Department of Neurobiology, Care Sciences and Society, Karolinska Institutet, SE-171 77 Stockholm, Sweden; Division of Neurogeriatrics, Department of Neurobiology, Care Sciences and Society, Karolinska Institutet, SE-171 77 Stockholm, Sweden; Division of Affinity Proteomics, Department of Protein Science, KTH Royal Institute of Technology, SciLifeLab, SE-171 65 Stockholm, Sweden; Division of Clinical Geriatrics, Department of Neurobiology, Care Sciences and Society, Karolinska Institutet, SE-171 77 Stockholm, Sweden; Institute of Clinical Medicine, University of Eastern Finland, FI-70210 Kuopio, Finland; Division of Clinical Geriatrics, Department of Neurobiology, Care Sciences and Society, Karolinska Institutet, SE-171 77 Stockholm, Sweden; Theme Inflammation and Aging, Karolinska University Hospital, SE-171 76 Stockholm, Sweden; Division of Clinical Geriatrics, Department of Neurobiology, Care Sciences and Society, Karolinska Institutet, SE-171 77 Stockholm, Sweden; Division of Clinical Geriatrics, Department of Neurobiology, Care Sciences and Society, Karolinska Institutet, SE-171 77 Stockholm, Sweden; Division of Affinity Proteomics, Department of Protein Science, KTH Royal Institute of Technology, SciLifeLab, SE-171 65 Stockholm, Sweden; Division of Clinical Geriatrics, Department of Neurobiology, Care Sciences and Society, Karolinska Institutet, SE-171 77 Stockholm, Sweden; Theme Inflammation and Aging, Karolinska University Hospital, SE-171 76 Stockholm, Sweden; Ageing Epidemiology Research Unit (AGE), School of Public Health, Imperial College London, London W12 OBZ, United Kingdom; Institute of Public Health and Clinical Nutrition, University of Eastern Finland, FI-70211 Kuopio, Finland; Division of Affinity Proteomics, Department of Protein Science, KTH Royal Institute of Technology, SciLifeLab, SE-171 65 Stockholm, Sweden; Division of Clinical Geriatrics, Department of Neurobiology, Care Sciences and Society, Karolinska Institutet, SE-171 77 Stockholm, Sweden; Division of Neurogeriatrics, Department of Neurobiology, Care Sciences and Society, Karolinska Institutet, SE-171 77 Stockholm, Sweden; Ageing Epidemiology Research Unit (AGE), School of Public Health, Imperial College London, London W12 OBZ, United Kingdom

**Keywords:** Alzheimer's disease, cognition, protein profiling, biosignature, biomarkers

## Abstract

Alzheimer’s disease and related dementias have a multifactorial aetiology and heterogeneous biology. The current study aims to identify different biological signatures in a deeply phenotyped memory clinic patient population. In this cross-sectional study, we analysed 49 pre-specified proteins using a multiplex antibody-based suspension bead array in 278 CSF samples from the real-world research database and biobank at the Karolinska University Hospital Memory Clinic, Solna, Sweden. Patients with a clinical diagnosis of subjective cognitive decline (*N* = 151), mild cognitive impairment (*N* = 61), Alzheimer’s disease (*N* = 47), or other diagnoses (*N* = 19; vascular dementias, alcohol-related dementia, unspecified dementias, or other amnesias) were included. Principal component analyses were performed, and resulting principal components (PCs) were tested for associations with clinical variables and Alzheimer’s disease biomarkers (CSF biomarkers beta-amyloid 42, beta-amyloid 42/40, phosphorylated tau 181, phosphorylated tau 181/beta-amyloid 42). PC 1 (explaining 52% of the variance between patients) was associated with the clinical Alzheimer’s disease CSF biomarkers beta-amyloid 42, phosphorylated tau 181, and total tau but not with Alzheimer’s disease-related neurodegeneration imaging markers, cognitive performance, or clinical diagnosis. PC 2 (explaining 9% of the variance) displayed an inflammatory profile with high contributions of chitinase 3 like 1 (CHI3L1) and triggering receptor expressed on myeloid cells 2 (TREM2) and significant correlation to CSF free light chain kappa. In contrast to PC 1, PC 3 (explaining 5% of the variance) showed associations with all the clinical Alzheimer’s disease CSF biomarkers, the imaging markers, cognitive impairment and clinical diagnosis. Serpin family A member 3 (SERPINA3), chitinase 1 (CHIT1), and neuronal pentraxin 2 (NPTX2) contributed most to PC 3. PC 4 (explaining 4% of the variance) exhibited an inflammatory profile distinct from PC 2, with the largest contributions from TREM2, leucine-rich alpha-2-glycoprotein 1 (LRG1) and complement C9. The component was associated with peripheral inflammation. We found that CSF protein profiles in a memory clinic cohort reflect molecular differences across diagnostic groups. Our results emphasize that real-world memory clinic patients can have different ongoing biological processes despite receiving the same diagnosis. In the future, this information could be utilized to identify patient endotypes and uncover precision biomarkers and novel therapeutic targets.

## Teaser

Alanko *et al*. showed that a multiplex panel of brain-derived proteins in the CSF reflects biological heterogeneity in a real-world memory clinic cohort. The biosignatures were associated with the Alzheimer’s disease CSF biomarker profiles of ATN (amyloid, phosphorylated tau and neurodegeneration pathology), yet only to a small extent with clinical diagnosis, cognition and imaging biomarkers.

## Graphical Abstract

**Graphical Abstract:**
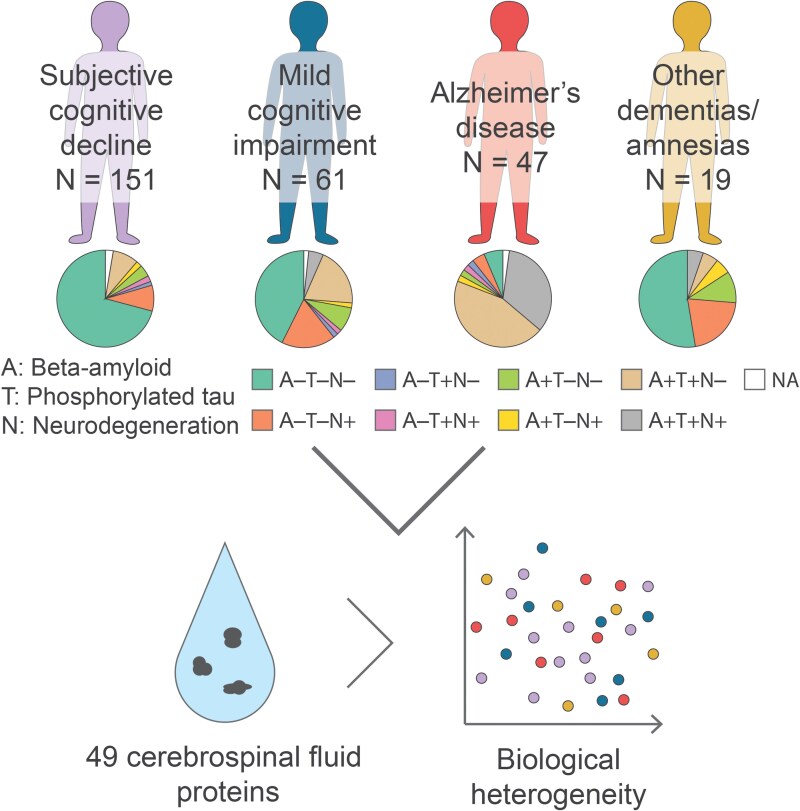

## Introduction

Alzheimer’s disease has a multifactorial aetiology, and the disease often co-exists with other brain pathologies, such as cerebrovascular, Lewy body and/or TAR DNA-binding protein 43 pathologies, resulting in heterogeneity in biological signatures among patients within the Alzheimer’s disease continuum.^1-5^ In general, to capture patients in the Alzheimer’s disease continuum, they are stratified based on the ATN criteria.^6,7^ While these criteria are useful to distinguish patients with the pathological changes that define Alzheimer’s disease, including beta-amyloid (Aβ) accumulation and tau phosphorylation, they are insufficient in identifying the variety of other underlying differences between patients that may be relevant for disease progression. Furthermore, despite recent breakthroughs and advancements in Alzheimer’s disease anti-amyloid drug development,^8-10^ there are disparities among the eligible patients in how well they respond to the treatment.^11^ There is a risk that the treatment effects may not be clinically relevant enough due to other underlying co-pathologies that continue to progress regardless of the anti-amyloid treatment. Disease-modifying therapies towards other pathologies and processes, such as inflammation or synaptic plasticity, are therefore essential, particularly for Alzheimer’s disease patients who are not eligible for the anti-amyloid treatments.^12,13^ It is therefore important to also look beyond the traditional Alzheimer’s disease biomarkers to recognize both connected and independent processes that may contribute to clinical symptoms. In the recently revised Alzheimer’s disease diagnostic and staging criteria, biomarkers of processes related to Alzheimer’s disease pathophysiology (neuronal injury and inflammation) and of non-Alzheimer’s disease co-pathologies (vascular brain injury and α-synuclein) have been included to account for the heterogeneity in biomarker profiles among patients.^7^ An improved understanding of the biological differences and similarities between patients could generate avenues for the development of drugs aimed at specific biosignatures or endotypes rather than specific diagnostic groups.

The current study aims to investigate the biological heterogeneity within a deeply phenotyped memory clinic patient population using a targeted multiplex protein assay on CSF samples. The population was a real-world cohort, i.e. it was not filtered based on ATN categories, diagnosis, or other characteristics to enable identification of biological signatures of patients that are independent of predefined stratification, and it was not part of any ongoing clinical study. The analysed proteins were pre-selected mainly based on findings in our previous studies as potential markers of Alzheimer’s disease and neurodegeneration.^14-19^ The panel was primarily composed of proteins expressed in the brain but also included some proteins that are expressed in other tissues. By enhancing the knowledge of the biological characteristics—whether related or unrelated to a clinical diagnosis and/or disease—of patients in a memory clinic, we can envision the identification of more personalized prevention and/or treatment strategies.

## Materials and methods

### Ethics approval and consent to participate

The GEDOC research database and biobank and the current study have been approved by the Swedish Ethical Review Authority (Dnr 2011/1987-31/4 and 2020-06484, respectively). All patients included in GEDOC have provided their informed consent.

### Cohort description

#### Study population

For this cross-sectional study, samples were retrieved from patients at the Karolinska University Hospital Medical Unit Aging Memory Clinic in Solna, Stockholm, Sweden. All available CSF samples collected from consecutive patients between April 2018 and March 2021 were included in the analysis (*N* = 288). Hence, the cohort was a real-world clinical cohort that consisted of patients with different diagnoses and Alzheimer’s disease biomarker profiles where we did not apply any exclusion criteria; however, nine patients were removed from the analyses due to high CSF erythrocyte values indicating blood contamination of the CSF samples and an additional patient due to low bead count in the multiplex CSF analysis (described below). The basic demographical characteristics of the population included in the main analyses (*N* = 278) are presented in Table 1. The procedures at the memory clinic are described in detail in a previous publication.^12^ Briefly, the clinical examination follows the Swedish national guidelines established by the Swedish Board of Health and Welfare^20^ and the diagnoses are set by consensus in a multidisciplinary team. The diagnoses are decided based on clinical evaluation and supported by medical history, brain imaging results, fluid biomarker values and cognitive assessments. Therefore, for example, poor performance in cognitive tests can be evaluated as independent of neurodegenerative diseases. The Diagnostic and Statistical Manual of Mental Disorders-V criteria are used as the basis for the diagnoses, and the diagnoses are coded according to ICD-10 coding. For the current study, patients were categorised into four main diagnostic groups: subjective cognitive decline (SCD), mild cognitive impairment (MCI), Alzheimer’s disease (AD), and other dementias/amnesias (Other). Patients categorised in the diagnostic group ‘Other’ had primary, ICD-coded diagnoses of vascular dementias, alcohol-related dementia, unspecified dementias, or other amnesias. Five established cognitive tests have been used to objectively evaluate the cognitive performance of the patients: the fourth edition (WAIS IV) of the Digit Symbol-Coding test (KOD), Mini Mental State Examination (MMSE), Montreal Cognitive Assessment (MoCA), Rey Auditory Verbal Learning Test total score (RAVLT) and Rey Complex Figure memory test (RCF).

**Table 1:** Demographics of the study cohort

	All	SCD	MCI	AD	Other
*N*	278	151	61	47	19
Age (years)^a,b^	60 (33–80)	58 (33–74)	61 (39–71)	62 (51–80)	62 (54–74)
Sex F/M (%)^c^	57.9/42.1	64.9/35.1	49.2/50.8	59.6/40.4	26.3/73.7
*APOE4* carrier/non-carrier/missing (%)^d^	39.9/51.4/8.6	32.5/57.6/9.9	44.3/49.2/6.5	55.3/36.2/8.5	47.4/47.4/5.2
ATN categories (%)^e^					
A–T–N–	52.5	70.9	42.6	6.4	52.6
A–T–N+/A–T+N–/ A–T+N+	10.8/1.4/1.8	8.6/1.3/2.0	18.0/1.6/1.6	4.3/2.1/2.1	21.1/0/0
A+T–N–/A+T+N–/ A+T–N+	5.0/16.9/2.2	4.0/8.6/2.0	8.2/19.7/1.6	2.1/44.7/2.1	10.5/5.3/5.3
A+T+N+	7.2	0	4.9	34.0	5.3
Missing	2.2	2.6	1.6	2.1	0
CSF markers					
Aβ42 (pg/mL)^a,b^	935 (248–2200)	1070 (307–2200)	791 (304–2070)	497 (248–1580)	935 (386–1570)
Aβ42/40 (x 10)^a,b^	0.92 (0.26–1.34)	0.99 (0.28–1.29)	0.87 (0.28–1.20)	0.48 (0.26–1.07)	0.91 (0.53–1.34)
p-tau181 (pg/mL)^a,b^	41 (14–190)	35 (15–120)	43 (14–120)	82 (19–190)	38 (19–100)
p-tau181/Aβ42^a,b^	0.036 (0.018–0.444)	0.030 (0.018–0.177)	0.043 (0.019–0.252)	0.172 (0.034–0.444)	0.036 (0.021–0.146)
t-tau (pg/mL)^a,b^	282 (75–1290)	236 (75–907)	285.5 (75–1290)	571 (151–1200)	285 (150–1010)
NfL (pg/mL)^a,b^	830 (300–9660)	670 (300–3960)	900 (390–5700)	1195 (730–9660)	1090 (510–9050)
Albumin quotient^a,f^	5.9 (2.0–35.0)	5.6 (2.3–14.4)	6.1 (2.0–35.0)	6.7 (2.6–12.8)	8.0 (4.0–18.0)
Protein concentration (µg/mL)^a,f^	297 (131–1245)	278 (147–693)	328 (131–1245)	322 (166–630)	359 (224–841)
MRI					
AD signature thickness^a,b^	2.64 (2.19–2.94)	2.66 (2.48–2.85)	2.65 (2.39–2.94)	2.48 (2.19–2.77)	2.56 (2.45–2.83)
Cortical thickness^a,b^	2.38 (2.03–2.58)	2.40 (2.21–2.55)	2.39 (2.19–2.58)	2.28 (2.03–2.56)	2.33 (2.20–2.46)
Hippocampal volume^a,b^	3846 (2184–5043)	4004 (3168–5043)	3862 (2844–4863)	3330 (2185–3876)	3661 (2234–4629)
WM hypointensities^a,b^	1460 (–1461–28 237)	1080 (–1461–28 237)	1586 (–193–23 659)	2440 (412–19 810)	3599 (883–27 837)
Cognition					
MMSE^a,b^	27 (14–30)	28 (16–30)	27 (14–30)	21 (14–30)	22 (18–28)
MoCA^a,b^	24 (5–30)	26 (13–30)	23 (5.5–29)	16 (5–24)	18 (12–27)
RAVLT^a,b^	44 (3–69)	49 (27–69)	36 (14–62)	25 (3–44)	25 (12–36)
RCF^a,b^	14 (0–33)	18 (2–33)	14 (0.5–25)	6 (0–11)	6.25 (0–14)
KOD^a,b^	49 (1–103)	56 (23–103)	45 (20–92)	25 (1–58)	34 (8–64)

^a^Values are given as median (min–max).

^b^Kruskal–Wallis rank sum test *P*-value <0.0001 when comparing the four different diagnostic groups.

^c^Pearson’s *χ*² test *P*-value <0.01 when comparing the four different diagnostic groups.

^d^Pearson’s *χ*² test *P*-value <0.05 when comparing the four different diagnostic groups.

^e^Pearson’s *χ*² test *P*-value <0.0001 when comparing the four different diagnostic groups.

^f^Kruskal­Wallis rank sum test *P*-value <0.05 when comparing the four different diagnostic groups.

Aβ, beta-amyloid; AD, Alzheimer’s disease; ATN categories, (A) beta-amyloid, (T) p-tau, (N) neurodegeneration (+) positive or (–) negative; *APOE4*, Apolipoprotein E allele E4; CSF, cerebrospinal fluid; KOD, Digit Symbol-Coding test; MCI, mild cognitive impairment; MMSE, Mini Mental State Examination; MoCA, Montreal Cognitive Assessment; MRI, magnetic resonance imaging; NfL, neurofilament light; p-tau, phosphorylated tau; RAVLT, Rey Auditory Verbal Learning Test; RCF, Rey Complex Figure memory test; SCD, subjective cognitive decline; t-tau, total tau; WM, white matter.

#### Analysis of CSF Alzheimer’s disease biomarkers

CSF Alzheimer’s disease biomarkers were measured at the Karolinska University Hospital Laboratory using commercially available enzyme-linked immunosorbent assays (ELISAs). Aβ40, Aβ42, p-tau181 and t-tau were measured using Innotest AMYLOID (1–40), Innotest AMYLOID (1–42), Innotest Phosphotau (181P) and Innotest hTAU-Ag (all from Fujirebio), respectively, until 22 August 2019. Later, samples were analysed with the Lumipulse G-series chemiluminescent enzyme immunoassay (Fujirebio Europe). Despite the change in measurement platform, we did not observe that our results would have been platform- or timepoint-dependent (data not shown). NfL was measured using an NF-Light ELISA (10-7001, Uman Diagnostics). Albumin concentrations in both CSF and serum were measured using the BN ProSpec/Atellica NEPH platform (Siemens Healthineers). Patient categorization into the ATN framework^6^ was based on (A) CSF Aβ42/40 (× 10) with a cut-off value of 0.68, (T) CSF p-tau181 with a cut-off value of 56.5 pg/mL, and (N) CSF NfL cut-off values of 890 pg/mL (age 60 years or younger) and 1850 pg/mL (age 61 years or older) (Table 1). Cut-offs were provided by the laboratory/manufacturer. Other clinical laboratory blood tests were measured at the Karolinska University Hospital Laboratory according to standard procedures. The following analytes were measured from blood, plasma, or serum samples: 25-OH vitamin D, alanine transaminase, alkaline phosphatase, aspartate aminotransferase (ASAT), calcium, carbohydrate-deficient transferrin, creatinine, erythrocyte sedimentation rate (SR), erythrocyte volume fraction, erythrocyte count, free light chain kappa (FLC-K), glutamyltransferase, haemoglobin, HbA1c, high-density lipoproteins cholesterol (HDL-C), homocysteine, International Normalized Ratio, kalium, leukocyte count, sodium, thrombocyte count, free thyroxine (T4), thyroid stimulating hormone, total cholesterol.

#### MRI

MRI scans were acquired on 3 Tesla GE Medical Systems Discovery MR750 scanners. We assessed brain morphology by T1-weighted 3D BRAVO sequence with slice thickness = 1 mm, repetition time = 8.16 ms and echo time = 3.18 ms. We processed the MRI through TheHiveDB system^21^ using the cross-sectional stream of FreeSurfer 7.3.2 (http://freesurfer.net/). Automatic region of interest parcellation and segmentation yielded morphological measures (thickness and volume) in cortical and subcortical structures.^22,23^ Resulting segmentations and estimations of intracranial volume were visually screened for quality control. We examined mean grey matter thickness, the mean thickness of Alzheimer’s disease signature regions,^24^ hippocampal volume and white matter (WM) hypointensity volume.^25^ The volume measures were adjusted for estimated intracranial volume using the residual approach.^26^ MRI measures were available only for a subset of patients (Total, *N* = 189; SCD, *N* = 97; mild cognitive impairment, *N* = 45; Alzheimer’s disease, *N* = 33; Other, *N* = 14).

### CSF protein analysis using antibody-based suspension bead array

For the multiplex protein detection, an antibody-based suspension bead array was used. The method has been described in detail previously.^18^ Shortly, 75 antibodies (Supplementary Table 1) against a pre-selected panel of 73 proteins were coupled to colour-coded, magnetic beads (MagPlex Microspheres, Luminex, TX, USA) by EDC/NHS crosslinking where one bead colour (ID) corresponded to one antibody. The majority of the antibodies (*N* = 72) were produced in rabbits, 69 of which were obtained from the Human Protein Atlas (https://www.proteinatlas.org/); two antibodies were produced in mice and one antibody was produced in goat. The antibody-coupled beads were then mixed to create the suspension bead array. CSF samples were biotin labelled (A39259, Thermo Scientific, MA, USA), heat treated at 56°C for 30 min and added to the bead array on a 384-well plate for an overnight incubation at RT. Finally, the samples were incubated with a streptavidin-conjugated fluorophore (SA10044, Invitrogen, MA, USA) and run in the Luminex Flexmap 3D instrument (Luminex Corp.). The read-outs were given as fluorescent intensities and data were obtained as median fluorescent intensity per bead ID and sample. Buffer, control beads and pooled CSF samples were included as internal controls.

### Data analysis

#### Quality control

All read-outs were assessed for quality and corrected for batch effect between sample plates and for delayed readout in the instrument based on sample position on the plate.^17,27^ For each antibody, more than 37 beads were required to be detected in a sample to be included in further analyses. All but one sample (<37 beads for 18 antibodies) passed this limit for all antibodies. Only antibodies with an inter-assay correlation of *rho* ≥ 0.7 were included in the downstream analyses. Moreover, in the case of two antibodies targeting the same protein, only the antibody with a stronger inter-assay correlation was included in the analyses. As a result, 49 proteins were included in the analyses (Supplementary Table 1).

#### Data adjustments

While the total protein concentration in CSF is an important marker for blood-brain barrier (BBB) dysfunction, other factors such as age, sex and CSF flow also influence the total protein concentration.^28,29^ These factors can influence protein levels unrelated to pathology; for instance, this has been seen in Alzheimer’s disease.^30^ Since our objective was to investigate protein levels independent of total protein concentration, the median fluorescent intensity data acquired for each protein was adjusted for total protein concentration measured using the Bradford Protein Assay (23200, Thermo Fisher, MA, USA). The correlation coefficient between total protein concentration and albumin quotient was *r* = 0.96 (Pearson’s correlation, *N* = 258). To normalize for total protein concentration, each analyte was adjusted by acquiring residuals from robust linear regression models (rlm, MASS), where the individual protein levels were considered outcome variables and total protein concentration the predictor. The residuals were further adjusted by adding the original median value across all samples for the respective protein. In case negative values remained after adding the median value, the adjusted values were further added by the minimum value (absolute value) + 2.

The distribution of the data for each protein was assessed visually by density plots and by the Shapiro-Wilk Normality Test (shapiro.test, stats), skewness (skewness, e1071) and kurtosis (kurtosis, e1071). Values over 4 SD from the mean for proteins with a kurtosis above 5 were considered outliers with a large impact on the kurtosis and were temporarily dropped. Next, all proteins that had a skewness of <−1 or >1 were considered non-normally distributed. These proteins were exported to Stata and zero-skewness log transformation (lnskew0) was applied to the protein variables. Proteins that have been log-transformed are indicated by ‘_ln’ after the protein abbreviation. Dropped protein values were added using the log transformation-generated equation if it did not largely impact the distribution of the data. If the dropped value was still a clear outlier, the dropped values were imputed to the maximum value of the remaining data (Supplementary Table 1). These measures were taken to attain normally distributed data. For the final data set, the skewnesses ranged from −0.74 (ITIH1) and 1.03 (TPPP) with a median skewness of 0.44.

#### Statistical analyses

All data handling, statistical analyses and visualization were performed using the R software (version 4.3.0), except for the log transformation (lnskew0 function) of selected protein values, which was done in the Stata software (version 17.0; StataCorp LLC, TX, USA). In R, the following packages were used: vroom, tidyverse, ggpubr, ggbeeswarm, matrixStats, rstatix, factoextra and RColorBrewer. Additional packages and used functions are indicated in parentheses. The principal component analysis (PCA) was done to analyse the dimensions of the selected proteins and their correlations to available clinical data and Alzheimer’s disease biomarkers. The PCA was run on a data set including all the patients (*N* = 278) and all the proteins (*N* = 49). Before running PCA, all protein values were centred and scaled to create Z-scores of the protein values (scale, base). PCA was performed using the ‘prcomp’ function (stats).

The four first principal components (PC) accounting for a cumulative variance of 70% (Table 2, Supplementary Table 2) were correlated with available clinical data and Alzheimer’s disease biomarkers to assess which clinical variables are reflected in the PCs. For all correlation analyses, Spearman’s rank correlation was used (cor, stats; cor.test, stats) and unadjusted *P*-values are reported. Age-adjusted correlations were analysed using partial Spearman’s rank correlation (partial_Spearman, PResiduals).^31^ Differences in categorical or dichotomous clinical characteristics between PCs were analysed using one-way ANOVA (anova_test, rstatix), Kruskal­ Wallis test (kruskal_test, rstatix) or two-tailed Student’s *t*-test (t_test, rstatix) when appropriate. Post hoc tests were performed with the two-sided Student’s *t*-test or Dunn’s test (dunn_test, rstatix) with FDR *P*-value adjustment when appropriate. Generally, *P*-values <0.05 were considered significant.

**Table 2:** Top 10 contributions (%) from principal component analysis

	PC 1		PC 2		PC 3		PC 4	
Eigenvalue	25.5		4.2		2.6		2.1	
Variance (%)	52.0		8.5		5.3		4.2	
Cumulative variance (%)	52.0		60.6		65.8		70.0	
	Protein	Contribution	Protein	Contribution	Protein	Contribution	Protein	Contribution
	OMG	3.6	CHI3L1_ln	12.7	SERPINA3	12.9	TREM2	15.3
	CHL1	3.5	CNP	12.2	CHIT1_ln	8.7	LRG1	11.6
	PAM	3.5	TNR	11.6	NPTX2	7.1	C9	11.2
	AMPH_ln	3.4	KNG1	11.1	AQP4	6.6	TNR	6.5
	PTPRN2	3.4	TPP1_ln	9.2	IGFBP2	5.5	TPPP_ln	5.8
	SNCA	3.4	TREM2	6.1	NPTXR_ln	4.5	SCG3	5.7
	PEBP1	3.4	ITIH1	6.1	VGF	3.9	CNP	5.4
	NCAN	3.4	C9	5.6	VCAM1	3.7	TPP1_ln	5.2
	NRCAM_ln	3.3	TPPP_ln	5.4	NPTX1	3.4	KNG1	4.8
	TMEM132D_ln	3.3	LRG1	4.7	ARPP21	3.4	SERPINA3	4.2

## Results

### Protein contributions to the different principal components

**Figure 1:**
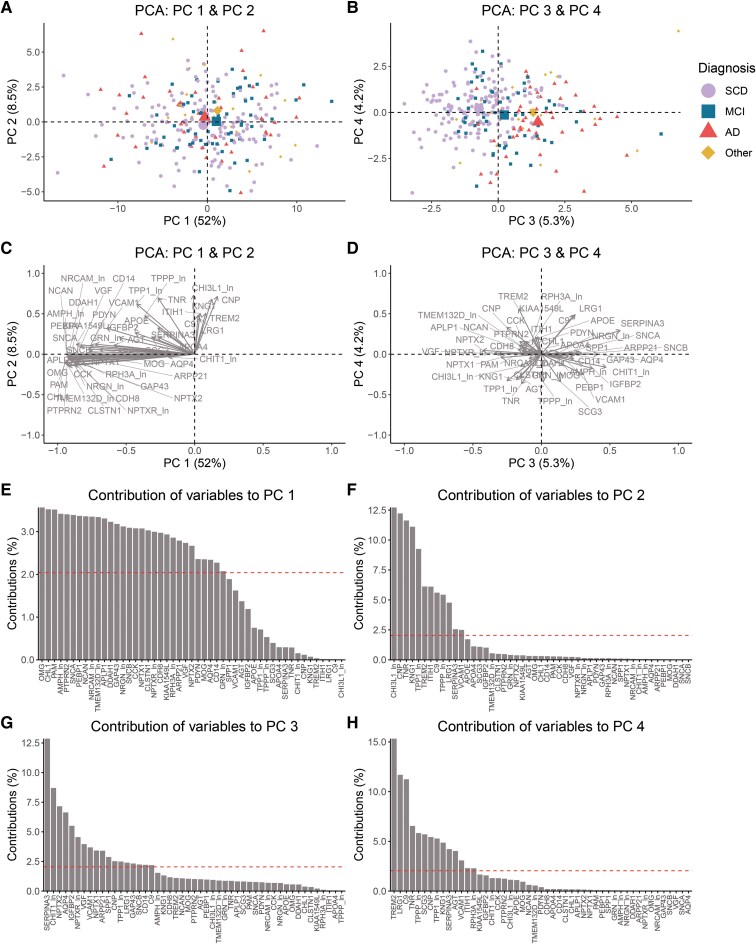
**The PCA reveals variance between patients based on CSF protein profile.** PCA was performed using centred and scaled protein level data from 49 proteins measured in CSF samples from a memory clinic cohort with 278 patients. Biplots of (**A**) PCs 1 and 2, and (**B**) 3 and 4 where the large points indicate the mean for each diagnostic group and each small data point represents a patient (SCD *N* = 151; MCI *N* = 61; AD *N* = 47; Other *N* = 19). The direction and strengths of variable contributions are visualized in correlation circle biplots for (**C**) PC 1 and PC 2, and (**D**) PC 3 and PC 4. Each protein is represented by an arrow. (**E–H**) Protein contributions (%) to each of the four PCs. The red dashed line indicates the level (2.04%) if all proteins contributed equally. A list of the protein names is given in Supplementary Table 1. AD, Alzheimer’s disease; MCI, mild cognitive impairment; Other, other dementias/amnesias; SCD, subjective cognitive decline.

To reduce the complexity of the protein data and to identify proteins that reflect the biological variance of the patients as determined by their CSF protein levels, we performed a PCA. The analysis resulted in four components together explaining 70% of the variance (Table 2, Supplementary Table 2). The PCA components and the contribution of variables are shown in Fig. 1. The first component (PC 1), which alone accounted for 52.0% of the variation among the patients, was not associated with the diagnosis of the patients (Fig. 1A, Supplementary Fig. 1A) but was able to differentiate the patients according to their ATN status. Groups positive for p-tau pathology (T+) had on average lower PC 1 values than p-tau negative (T−) groups with an exception for the A+T+N+ group that had a PC 1 level comparable to the A–T–N– (Supplementary Fig. 2A,C). The variance observed for PC 1 was influenced by a large group of 30 proteins all with a contribution between 2.1% and 3.6% (Fig. 1E). The correlation circle biplot indicates that these proteins are highly positively intercorrelated (Fig. 1C).

PC 2 (8.5% variance explained) was more strongly driven by a subset of specific proteins, where chitinase 3 like 1 (also known as YKL-40) had the largest contribution of 12.7%, followed by CNP 12.2%, TNR 11.6%, KNG1 11.1% and TPP1 9.2% (Fig. 1F, Table 2). The component was further influenced by TREM2 (6.1%), ITIH1 (6.1%), C9 (5.6%) and LRG1 (4.7%). Like PC 1, there was no indication of PC 2 either being connected to the clinical diagnosis, and here no differences between ATN categories were observed (Supplementary Fig. 2A,C).

Along the PC 3 (5.3% variance explained) axis, on the other hand, the patients were spread based on the clinical diagnosis and differences between SCD and the other diagnostic groups were significant (Fig. 1B, Supplementary Fig. 1A). Although with some overlap, the A–T–N– patients on average deviated from the A+T+N+ and A+T+N– groups (Supplementary Fig. 2B,C). Serpin family A member 3 (SERPINA3) had the strongest impact on the component with a contribution of 12.9%, followed by chitinase 1 8.7%, NPTX2 7.1% and AQP4 6.6% (Fig. 1G, Table 2). The proteins that accounted for 60% of the contribution to the component (*N* = 10) had different directionalities; contributions of SERPINA3, chitinase 1, AQP4, IGFBP2 (5.5%), VCAM1 (3.7%) and ARPP21 (3.4%) were opposing to those for NPTX2, NPTXR (4.5%), VGF (3.9%) and NPTX1 (3.4%; Fig. 1D).

Between the 3 first components, there were no overlaps between the top 10 contributing proteins. On the contrary, 8 out of the top 10 contributions for PC 4 (4.2%; Table 2) were also included in the top 10 proteins of PC 2. PC 4 was most strongly influenced by TREM2 (15.3%), followed by LRG1 (11.6%) and C9 (11.2%) (Fig. 1H). In contrast to PC 2, where all the top proteins had similar directionality (Fig. 1C), in PC 4 the directions of the contributions varied (Fig. 1D). Both for the oligodendrocytic and the peripheral markers there were proteins with negative (TNR and TPPP, and KNG1, respectively) and positive (CNP and LRG1 and C9, respectively) directions. PC 4 was significantly different between SCD and Alzheimer’s disease groups (Supplementary Fig. 1A) but the component was not related to ATN (Supplementary Fig. 2B,C).

### Associations between principal components and clinical variables

**Figure 2:**
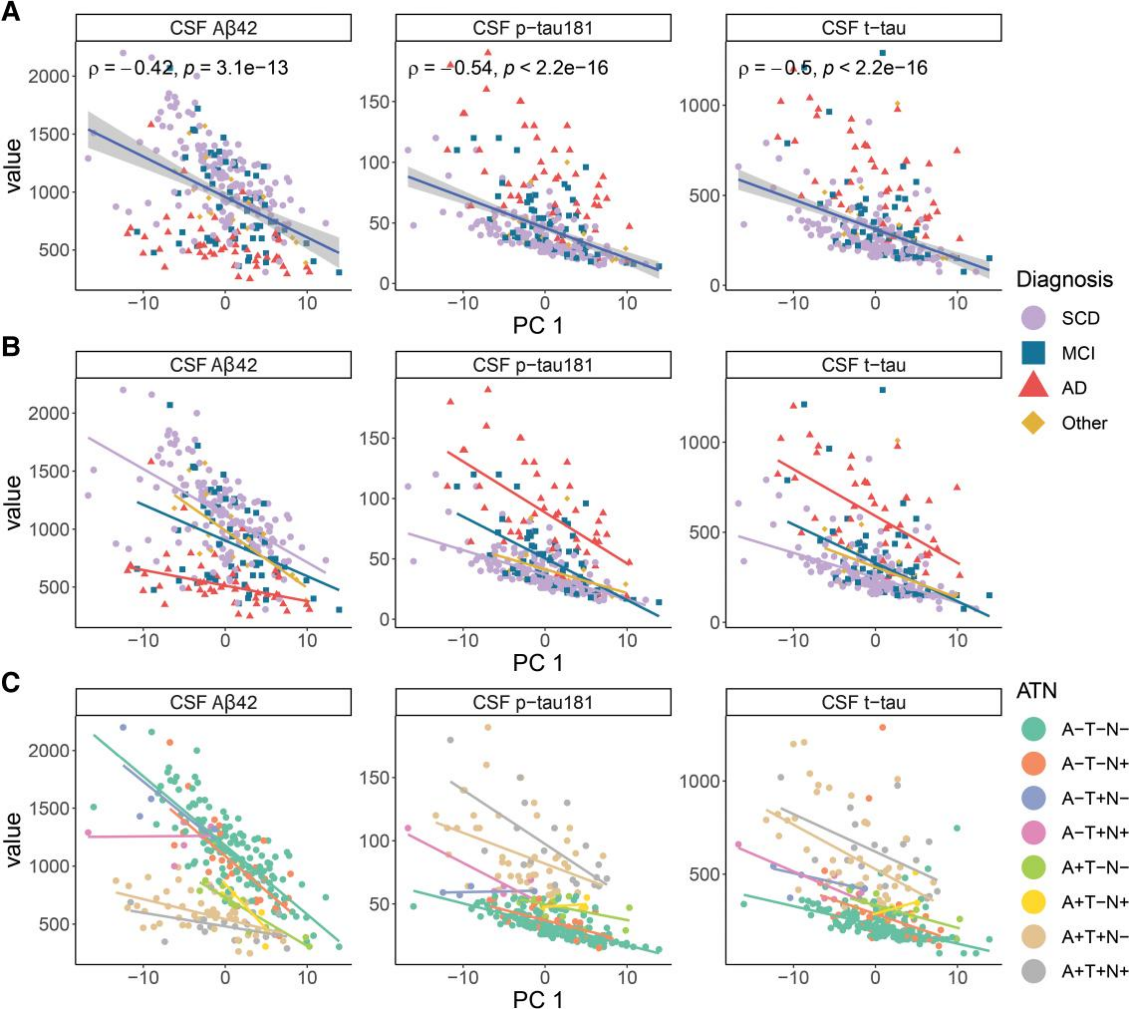
**PC 1 is correlated with three Alzheimer’s disease markers.** (**A**) Significant correlations (Spearman’s rank correlation) were observed between PC 1 and Aβ42 (pg/mL; *N* = 277), p-tau181 (pg/mL; *N* = 277) and t-tau (pg/mL; *N* = 277). (**B**) When stratified by diagnosis [SCD *N* = 151; MCI *N* = 60; AD *N* = 47; Other *N* = 19], the correlations remained significant (Supplementary Table 3B) and had the same directions. (**C**) Stratification by ATN category (A–T–N– *N* = 146; A–T–N+ *N* = 30; A–T+N– *N* = 4; A–T+N+ *N* = 5; A+T–N– *N* = 14; A+T–N+ *N* = 6; A+T+N– *N* = 47; A+T+N+ *N* = 20) revealed similar correlations between most of the categories. Only correlations with a significance level of *P* < 0.0001 (unadjusted) are shown and the plotted trendlines were acquired by robust linear regressions. Each data point represents a patient and is annotated by the diagnostic group or ATN category. Aβ, beta-amyloid; AD, Alzheimer’s disease; ATN categories, (A) beta-amyloid, (T) p-tau, (N) neurodegeneration (+) positive or (−) negative; MCI, mild cognitive impairment; Other, other dementias/amnesias; p-tau, phosphorylated tau; SCD, subjective cognitive decline; t-tau, total tau.

After determining the biological variance based on our measured proteins within the whole cohort, we correlated PC levels with Alzheimer’s disease biomarkers, cognitive test scores, and laboratory markers taking into account clinical groups and ATN status. Further differences between sexes and *APOE4* carrier statuses with respect to each PC were analysed. As the component was associated with ATN categories, expectedly, PC 1 showed significant correlations with CSF Alzheimer’s disease biomarkers Aβ42, p-tau181 and t-tau levels as indicated by their significant negative correlations (Spearman’s rank correlation, unadjusted *P*-values <0.0001 and absolute *rho* > 0.40, Fig. 2A, Table 3). Further adjustment for age only had a minor impact on the correlations (*P*-value adj and *rho* adj in Supplementary Table 3A). When stratified by diagnosis, all the diagnostic groups individually had a moderate to strong correlation between the respective Alzheimer’s disease biomarker and PC 1 (Fig. 2B, Supplementary Table 3B). A similar pattern was observed when patients were stratified by ATN categories (Fig. 2C, Supplementary Table 3B). There were no differences in PC 1 values between females and males or *APOE4* carriers or non-carriers (Supplementary Fig. 1B,C).

**Table 3:** Correlations between principal components and clinical variables (absolute *rho* > 0.20)

*N*	Variable	Principal component	Spearman’s *rho*	*P*-value
277	CSF p-tau181 (pg/mL)	PC 1	–0.54	1.3E–22
277	CSF t-tau (pg/mL)	PC 1	–0.50	6.3E–19
277	CSF Aβ42 (pg/mL)	PC 1	–0.42	3.1E–13
163	RCF	PC 1	–0.22	4.7E–03
272	CSF NfL (pg/mL)	PC 3	0.53	7.5E–21
277	CSF p-tau/Aβ42	PC 3	0.51	2.2E–19
172	RAVLT	PC 3	–0.49	8.9E–12
278	Age	PC 3	0.49	6.7E–18
163	RCF	PC 3	–0.48	6.5E–11
277	CSF Aβ42 (pg/mL)	PC 3	–0.47	2.3E–16
189	WM hypointensities	PC 3	0.43	4.2E-10
162	KOD	PC 3	–0.42	2.2E–08
277	CSF Aβ42/40	PC 3	–0.41	6.2E–13
234	MoCA	PC 3	–0.41	5.1E–11
189	Hippocampal volume	PC 3	–0.41	5.4E-09
277	CSF t-tau (pg/mL)	PC 3	0.40	5.5E–12
277	CSF p-tau181 (pg/mL)	PC 3	0.37	2.8E–10
187	MMSE	PC 3	–0.34	1.6E–06
189	Cortical thickness	PC 3	–0.33	4.2E-06
189	AD signature thickness	PC 3	–0.31	1.3E-05
241	Plasma homocysteine (µmol/L)	PC 3	0.28	9.4E–06
240	Plasma/serum FLC-K (mg/L)	PC 3	0.27	2.4E–05
240	Systolic blood pressure	PC 3	0.24	2.0E–04
239	Blood erythrocyte SR (mm)	PC 4	0.34	7.9E–08
242	Serum albumin (g/L)	PC 4	–0.30	1.6E–06
243	Plasma albumin (g/L)	PC 4	–0.28	1.1E–05
242	Blood haemoglobin (g/L)	PC 4	–0.26	3.3E–05
245	Blood erythrocyte volume fraction	PC 4	–0.24	1.1E–04
245	Blood erythrocytes (×10^12^/L)	PC 4	–0.21	1.2E–03

Aβ, Beta-amyloid; AD, Alzheimer’s disease; CSF, cerebrospinal fluid; FLC-K, free light chain kappa; KOD, Digit Symbol-Coding test; MMSE, Mini Mental State Examination; MoCA, Montreal Cognitive Assessment; NfL, neurofilament light; p-tau, phosphorylated tau; RAVLT, Rey Auditory Verbal Learning Test; RCF, Rey Complex Figure memory test; t-tau, total tau; WM, white matter.

**Figure 3:**
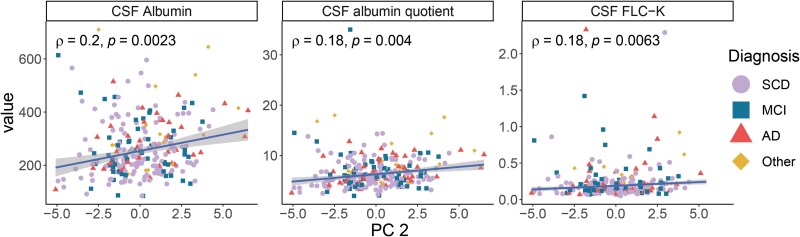
**PC 2 is weakly correlated with CSF albumin and FLC-K.** PC 2 was significantly correlated (Spearman’s rank correlation) with CSF albumin levels (mg/L; *N* = 241), albumin quotient (*N* = 258) and FLC-K (mg/L; *N* = 238). Only correlations with a significance level of *P* < 0.01 (unadjusted) are shown and the plotted trendlines were acquired by robust linear regressions. Each data point represents a patient and is annotated by the diagnostic group. Two data points were removed from the FLC-K plot for visual purposes: FLC-K 28 mg/L, PC 2 –1.44 (SCD, A–T–N+), FLC-K 11 mg/L, PC 2 2.54 (SCD, A–T–N–). AD, Alzheimer’s disease; FLC-K, free light chain kappa; MCI, mild cognitive impairment; Other, other dementias/amnesias; SCD, subjective cognitive decline.

In concordance with PC 2 not being associated with either diagnosis or ATN, the component did not show correlations to Alzheimer’s disease biomarkers or cognitive test scores. PC 2 was significantly yet weakly correlated with both CSF albumin (*rho* = 0.20, *P*-value = 0.002) and albumin quotient (*rho* = 0.18, *P*-value = 0.004; Fig. 3) indicating permeability of the BBB. PC 2 had a weak yet significant correlation of the clinical inflammatory marker CSF FLC-K (*rho* = 0.18, *P*-value = 0.006). After adjusting for age, the correlations remained the same (Supplementary Table 3A). There were no differences between sexes or *APOE4* carrier statuses (Supplementary Fig. 1B,C).

**Figure 4:**
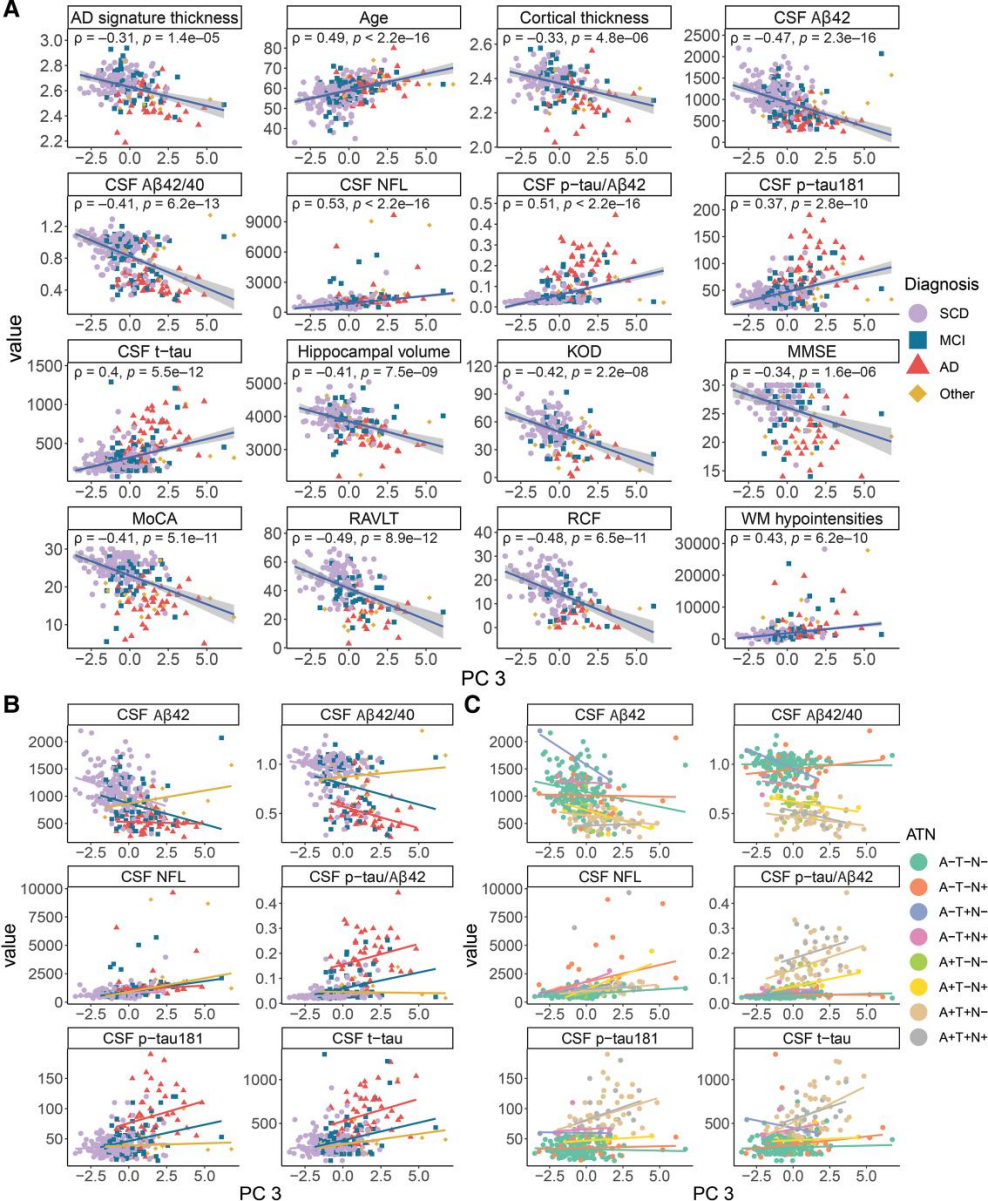
**PC 3 reflects Alzheimer’s disease pathology and cognitive impairment.** (**A**) Significant correlations (Spearman’s rank correlation) between PC 3 and all Alzheimer’s disease CSF biomarkers (Aβ42 *N* = 277; Aβ42/40 *N* = 277; p-tau/Aβ42 *N* = 277; p-tau181 *N* = 277), CSF markers of neuronal injury (NfL *N* = 272; t-tau *N* = 277), MRI measures of neurodegeneration (AD signature thickness, cortical thickness, hippocampal volume and WM hypointensities all *N* = 189), cognitive test scores (KOD *N* = 162; MMSE *N* = 187; MoCA *N* = 234; RAVLT *N* = 172; RCF *N* = 163) and age (*N* = 278). Units for the CSF variables are indicated in Table 1. (**B**) Correlations between CSF Alzheimer’s disease biomarkers and PC 3 were stratified by diagnosis [SCD *N* = 151 (NfL *N* = 147); MCI *N* = 60; AD *N* = 47 (NfL *N* = 46); Other *N* = 19]. The correlations remained significant only for some of the diagnostic groups (Supplementary Table 3B). (**C**) CSF Alzheimer’s disease biomarker correlations stratified by ATN category (A–T–N– *N* = 146; A–T–N+ *N* = 30; A–T+N– *N* = 4; A–T+N+ *N* = 5; A+T–N– *N* = 14; A+T–N+ *N* = 6; A+T+N– *N* = 47; A+T+N+ *N* = 20) displayed divergent relationships among the categories (Supplementary Table 3B). Only correlations with a significance level of *P* < 0.0001 (unadjusted) are shown and the plotted trendlines were acquired by robust linear regressions. Each data point represents a patient and is annotated by the diagnostic group or ATN category. Aβ; beta-amyloid; AD, Alzheimer's disease; ATN categories, (A) beta-amyloid, (T) p-tau, (N) neurodegeneration (+) positive or (−) negative; KOD, Digit Symbol-Coding test; MCI, mild cognitive impairment; MMSE, Mini Mental State Examination; MoCA, Montreal Cognitive Assessment; NfL, neurofilament light; Other, other dementias/amnesias; RAVLT, Rey Auditory Verbal Learning Test; RCF, Rey Complex Figure memory test; p-tau, phosphorylated tau; SCD, subjective cognitive decline; t-tau, total tau; WM, white matter.

PC 3 was correlated with the CSF biomarkers generally used for biologically defining Alzheimer’s disease (Aβ42, *P*-tau181, t-tau, Aβ42/40, p-tau/Aβ42) but also with the cognitive performance of the patients (MMSE, MoCA, KOD, RCF, RAVLT) and MRI measures (WM hypointensities, hippocampal volume, cortical thickness, Alzheimer’s disease signature thickness; Fig. 4A, Table 3). PC 3 was further strongly correlated with the neuronal injury marker CSF NfL (*rho* = 0.53, *P*-value < 0.0001). Still, PC 3 was significantly correlated with age and thus the other significant correlations became weaker after age adjustment (Supplementary Table 3A). For PC 3, in contrast to PC 1, patients were distributed along both the PC and the Alzheimer’s disease biomarker axes by the severity of cognitive decline reflected in the clinical diagnosis (Fig. 4A). When stratified by diagnosis, CSF Aβ42 was not correlated to PC 3 in the Alzheimer’s disease group, and the correlation between PC 3 and p-tau181, t-tau, and Aβ42/40 in the SCD group was non-significant or only weak (Fig. 4B, Supplementary Table 3B). The Other group (other dementias/amnesias) had significant correlations between PC 3 and CSF NfL and t-tau but not CSF Alzheimer’s disease biomarkers. When stratified by the ATN categories, the A–T–N– group had weak but significant correlations for CSF Aβ42, NfL and p-tau/Aβ42 (Fig. 4C, Supplementary Table 3B). In addition to variables indicating Alzheimer’s disease profile, we observed correlations with other clinical markers. PC 3 had weak but significant correlations (*P*-value < 0.0002) to plasma homocysteine (*rho* = 0.28), blood FLC-K (*rho* = 0.27) and systolic blood pressure (*rho* = 0.24; Table 3). While there were no differences between sexes, *APOE4* carriers had slightly higher PC 3 values than non-carriers, although this difference did not reach significance (Supplementary Fig. 1B,C).

**Figure 5:**
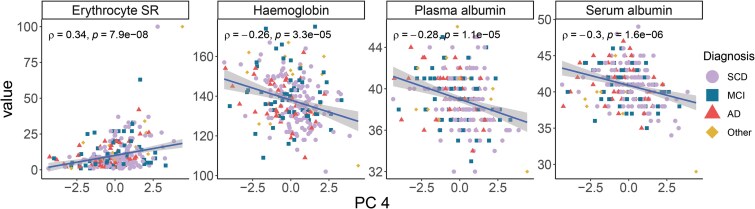
**PC 4 is associated with markers of peripheral inflammation.** PC 4 was significantly correlated (Spearman’s rank correlation) with erythrocyte SR (mm; *N* = 239), haemoglobin (g/L; *N* = 242) and both plasma and serum albumin (g/L; *N* = 243 and 242, respectively). Only correlations with a significance level of *P* < 0.0001 (unadjusted) are shown and the plotted trendlines were acquired by robust linear regressions. Each data point represents a patient and is annotated by the diagnostic group. AD, Alzheimer’s disease; MCI, mild cognitive impairment; Other, other dementias/amnesias; SCD, subjective cognitive decline; SR, sedimentation rate.

Although several driving proteins of PC 4 were shared with PC 2 (Table 2) there were differences in the direction of contributions between these components. Consequently, PC 4 showed correlations with other clinical markers (Fig. 5, Table 3). PC 4 was significantly correlated with blood erythrocyte SR (*rho* = 0.34), serum and plasma albumin (*rho* = −0.30 and −0.28, respectively), haemoglobin (*rho* = −0.26), blood erythrocyte volume fraction (haematocrit, *rho* = −0.24) and plasma creatinine (*rho* = −0.20; Fig. 5, Table 3). Age adjustment did not alter these correlations (Supplementary Table 3A). Moreover, women had on average higher PC 4 values than men, while *APOE4* carriers had on average lower, although not significantly, PC 4 values than non-carriers (Supplementary Fig. 1B,C).

## Discussion

Understanding the differences and similarities of disease-related processes between patients with similar clinical diagnoses can guide towards more personalized treatment and prevention strategies. Here, we report that a panel of brain-derived proteins captures biological heterogeneity in a real-world memory clinic cohort that has not been selected based on clinical characteristics or biomarker profiles, such as diagnosis or ATN status. We chose to study a panel of proteins known to be associated with Alzheimer’s disease and its biomarkers to extend the knowledge about the heterogeneity in protein profiles relevant to this disorder.^14,15^ We show that the panel reflects variances in Alzheimer’s disease biomarker levels and to a lesser extent ongoing Alzheimer’s disease pathological processes.

To understand which of the 49 proteins in the panel contributed most to explain the biological variance between the patients, a dimensionality reduction was performed using PCA. The homogeneity between contributions in PC 1 indicated that no single protein/small group of proteins was extensively contributing to the variation between patients, i.e., no association between specific cell type or function was found for PC 1. Many of the 30 proteins with a contribution larger than 2.0% are expressed in the brain (based on the transcriptomic data from the Human Protein Atlas v. 23^32^) and they are implicated in diverse processes.

The biological variance in PC 1 was not associated with the clinical diagnosis despite levels of some of these intercorrelated proteins, such as GAP43, AMPH, PTPRN2 and SNCB, having been observed to be different between diagnostic and/or ATN groups.^14,17^ Since we in our analyses observed that low PC 1 values were strongly related to a T+ category, we explored whether Alzheimer’s disease biomarker levels could account for a combined influence on PC 1. The biological variation between the patients in our cohort was indeed reflected in CSF Alzheimer’s disease biomarkers Aβ42, p-tau181 and t-tau. We have previously shown that p-tau181 is positively correlated with a majority of the proteins of our panel, irrespective of ATN status,^17^ which is in line with the differences seen between T− and T+ groups in PC 1. However, considering the lack of association between PC 1 and diagnosis, cognitive impairment, or MRI measures reflecting neurodegeneration, we postulate that PC 1 displays physiological rather than pathological associations to CSF Alzheimer’s disease biomarkers among the patients. Similar physiological variability in CSF protein levels has recently been described by Karlsson *et al*.^33^ Although having differences in correlation slopes, all diagnostic and majority of the ATN groups individually had a moderate to strong correlation between PC 1 and the Alzheimer’s disease biomarkers.

In contrast to PC 1, the three following components had stronger contributions from specific proteins or groups of proteins. Based on the contributions, PC 2 could be considered to be a component reflecting diagnosis-independent inflammation, oligodendrocyte functioning and BBB integrity. The main drivers were proteins previously shown to be involved in inflammatory processes (TREM2^30,34^), proteins relevant for oligodendrocyte functioning (CNP, TNR, TPPP^35-39^) but also proteins enriched in the liver (KNG1, ITIH1, C9, LRG1^32^). CSF FLC-K has been used as a biomarker for multiple sclerosis,^40^ and both CSF albumin levels and albumin quotient reflect BBB permeability.^41^ Taken together, the inflammatory and BBB integrity characteristics were further corroborated by correlations between PC 2 and FLC-K, albumin levels and quotient.

PC 3, on the other hand, was influenced by astrocytic proteins and proteins involved in synaptic plasticity and functioning, many of which have previously been studied as Alzheimer’s disease biomarkers. The component also has the clearest association with diagnosis and ATN compared with the other investigated components. Among the most prominent contributors to PC 3 were the astrocytic proteins SERPINA3 and IGFBP2 which have been recognized as novel potential biomarkers in Alzheimer’s disease; SERPINA3 is implicated in Aβ aggregation and IGFBP2 may impact tau phosphorylation through insulin-like growth factors^42,43^ and both proteins have shown potential as biomarkers in predicting incident dementia.^44,45^ Another of the identified main contributors to PC 3 was the astrocytic AQP4, which is required for brain fluid homeostasis and was previously found to be elevated in CSF of Alzheimer’s patients.^14,46^ Chitinase 1, which was the second largest contributor to PC 3, has been observed to be involved in reactive gliosis and CSF levels of the protein are increased in Alzheimer’s disease.^34,47^ Also, the PC 3 proteins involved in synaptic plasticity and functioning (NPTXR, NPTX2, NPTX1, VGF) have been proposed as biomarkers of Alzheimer’s disease.^48-50^ Importantly, the astrocytic and synapse-related proteins had an opposing influence on PC 3. This finding is in line with previous research where decreased levels of these synaptic proteins^48-50^ but increased levels of astrocytic/gliosis proteins^14,44,45,47^ have been associated with cognitive impairment and Alzheimer’s disease.

PC 3 also had strong relationships with both the Alzheimer’s disease biomarkers and clinical characteristics, resulting in PC 3 being a component reflecting a typical Alzheimer’s-like profile. Prominently, PC 3 reflected Alzheimer’s disease pathology rather than the physiological associations to Aβ and tau that were observed for PC 1. The relation to Alzheimer’s disease pathology was further reinforced when stratified by diagnosis, as correlations between the clinical Alzheimer’s disease biomarkers and PC 3 were not observed for the Other group as was seen for PC 1. Similarly, when stratified by ATN, A–T–N– patients had none or only weak correlations between PC 3 and Alzheimer’s disease CSF biomarkers. Collectively, the astrocytic and synaptic proteins that contributed to PC 3 could together with the core Alzheimer’s disease biomarkers provide additional insight into biomarker profiles that are important in Alzheimer’s disease pathogenesis. These additional proteins could be considered as non-core biomarkers to further develop the AT(X)N system^51^ recently integrated into the revised Alzheimer’s disease diagnosis and staging criteria.^7^

Despite being driven by largely the same proteins as PC 2, in PC 4 the protein contributions had different directionalities resulting in distinctive correlations to the clinical markers. PC 4 correlated with clinical biomarkers associated with general inflammatory processes (SR and blood albumin levels), anaemia (haemoglobin, erythrocyte volume fraction and erythrocyte count) and kidney function (creatinine). CSF-measured TREM2 has previously been found to be associated with both central and peripheral inflammatory markers, including SR.^30^ TREM2, together with LRG1 and C9, the two proteins with the highest contribution after TREM2, have all been implicated in innate immunity.^30,52^ These data would suggest that PC 4 is related to peripheral inflammation,^30,52,53^ whereas PC 2 might rather reflect CNS inflammation. Furthermore, these two components are largely independent of the traditional Alzheimer’s disease biomarkers and characteristics, indicating that variation between patients at a memory clinic can be seen on several levels.

This study has several limitations. Firstly, the cohort is relatively small and groups are not age balanced which could create possible bias. Interpreting CSF results is challenging as both the flow rate and volume change during ageing.^28,54^ However, adjustment for age in the correlations did not change the relationships between the PCs and other variables, suggesting that our findings in CSF biosignatures are mostly independent of age. Importantly, we had not made any prior selection of the patients based on, for example, clinical diagnosis or Alzheimer’s disease pathology, as our objective was to evaluate heterogeneity in a population representing the broad spectrum of patients assessed at a memory clinic. The cohort thus included patients with variable demographical characteristics, including SCD patients falling into the Alzheimer’s disease continuum based on their ATN profile as well as Alzheimer’s patients having a negative ATN profile, which could have influenced the results. Moreover, some of the ATN groups were very small and thus our results are inadequate to make conclusions for these groups. Secondly, our study was cross-sectional thus not taking into account any longitudinal changes in disease progression. Future studies should investigate heterogeneity in longitudinal cohorts and, for example, evaluate the diagnostic potential of the main drivers of PC 3 in Alzheimer’s disease development.

Our discoveries emphasize the potential to further distinguish molecular signatures within memory clinic cohorts using selected protein panels. Our findings are in line with previous research indicating a large biological heterogeneity in Alzheimer’s disease. We revealed that the biological signatures based on a targeted panel of CSF proteins were independent of clinical diagnosis yet related to CSF Alzheimer’s disease biomarkers. While with the help of the ATN framework, it is possible to biologically define and stratify Alzheimer’s disease, there are additional biological elements identified herein that could be important for disease manifestation and progression. Understanding and acknowledging this biological heterogeneity can be essential when aiming for tailored and personalized prevention and treatment options as it might impact the efficacy of interventions. Our findings add to the understanding of biological differences between patients in a memory clinic population.

## Supplementary Material

fcaf078_Supplementary_Data

## Data Availability

The data and R/Stata codes generated and/or analysed during the current study are not publicly available but are available from the corresponding author at reasonable request.
