# Supplementary material for: Biological signatures in the Alzheimer’s continuum discriminate between diagnosis-related and -unrelated associations to ATN categories
Source: Brain Commun. 2025 Feb 21;7(2):fcaf078. doi: 10.1093/braincomms/fcaf078 (PMC11881062; doi:10.1093/braincomms/fcaf078)
Supplement: fcaf078_Supplementary_Data [file fcaf078_supplementary_data.zip › Supplementary Figures.pdf]

Biological signatures in the Alzheimer's continuum discriminate between diagnosis-related and -unrelated associations to ATN categories

Vilma Alanko[1,2], Sára Mravinacová[3], Anette Hall[1,4], Göran Hagman[1,5], Rosaleena Mohanty[1], Eric Westman[1], Peter Nilsson[3], Miia Kivipelto[1,5,6,7], Anna Månberg[3], Anna Matton[1,2,6]

Author affiliations:

1. Division of Clinical Geriatrics, Department of Neurobiology, Care Sciences and Society, Karolinska Institutet, Stockholm, Sweden
2. Division of Neurogeriatrics, Department of Neurobiology, Care Sciences and Society, Karolinska Institutet, Stockholm, Sweden
3. Division of Affinity Proteomics, Department of Protein Science, KTH Royal Institute of Technology, SciLifeLab, Stockholm, Sweden
4. Institute of Clinical Medicine, University of Eastern Finland, Kuopio, Finland.
5. Theme Inflammation and Aging, Karolinska University Hospital, Stockholm, Sweden
6. Ageing Epidemiology (AGE) Research Unit, Imperial College London, London, United Kingdom
7. Institute of Public Health and Clinical Nutrition, University of Eastern Finland, Kuopio, Finland

Correspondence to: Anna Matton
Full address: Karolinska vägen 37A, QA32, 171 64 Solna, Sweden
E-mail: anna.matton@ki.se

Running title: Biosignatures in AD relative to diagnosis

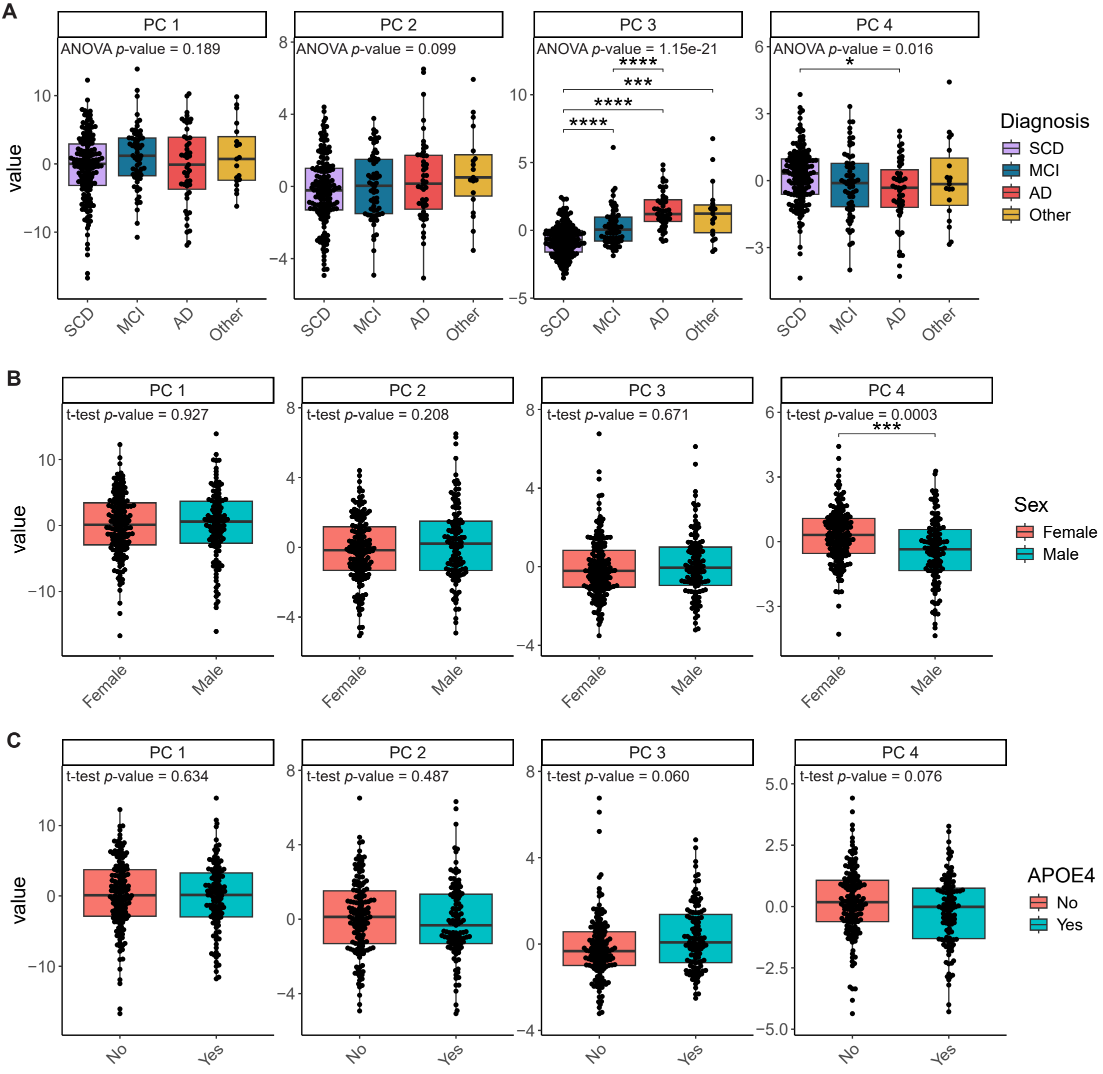

Supplementary Figure 1 Association between principal components (PC) and patient characteristics. Boxplots showing the differences between the (**A**) diagnostic groups (SCD N = 151; MCI N = 61; AD N = 47; Other N = 19), (**B**) sex (female N = 161, male N = 117), and (**C**) *APOE4* carrier status (carrier yes N = 111, no 143) for each PC's values where each data point represents a patient. The *p*-values have been FDR adjusted. (**A**) Post hoc tests were performed with the two-tailed Student's t-test with FDR *p*-value adjustment. **p* < 0.05, ****p* < 0.001, *****p* < 0.0001. Abbreviations: AD, Alzheimer's disease; *APOE4*, Apolipoprotein E allele E4; MCI, mild cognitive impairment; Other, other dementias/amnesias; SCD, subjective cognitive decline

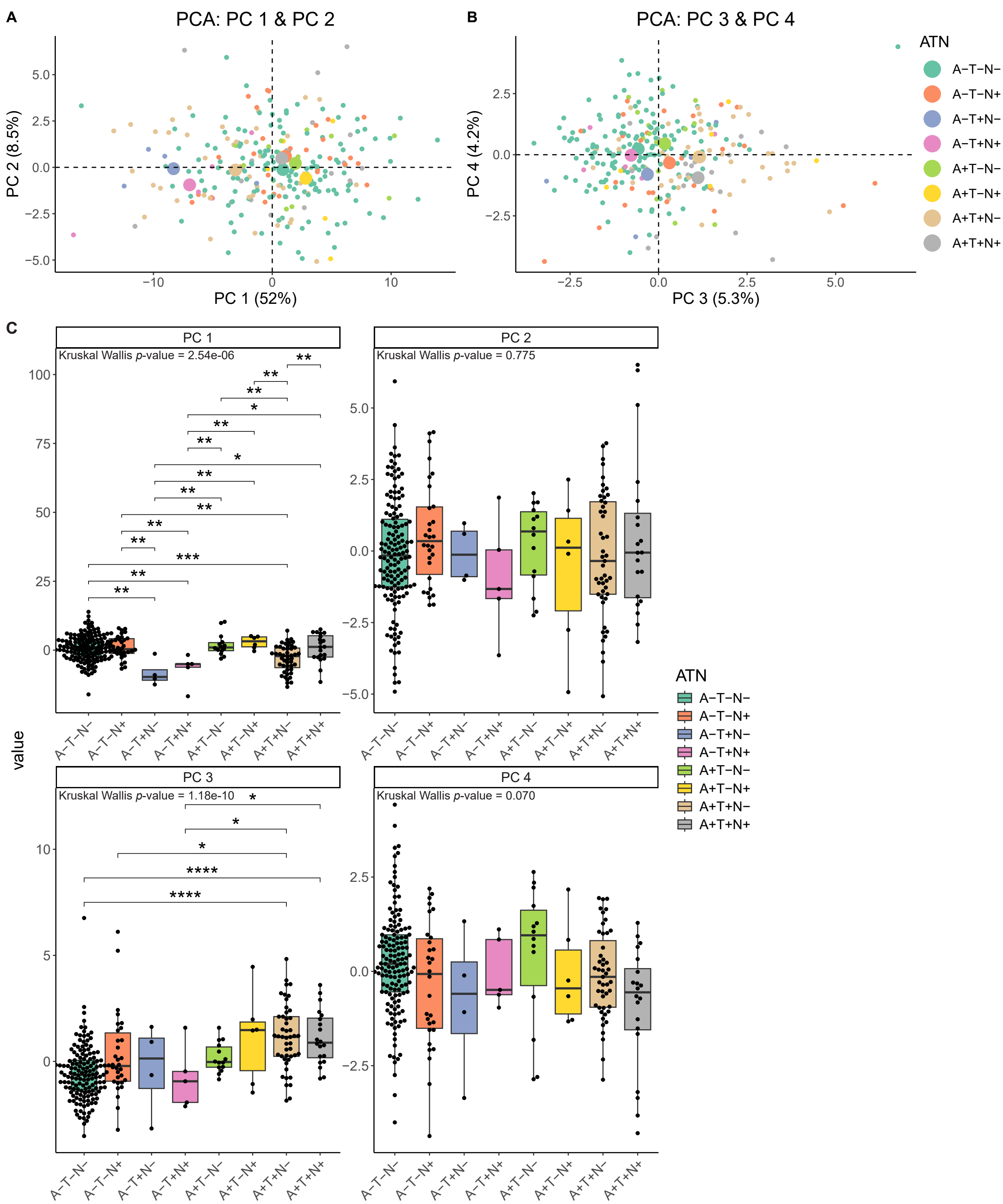

Supplementary Figure 2 Association between principal components (PC) and ATN categories. Biplots of (**A**) PCs 1 and 2, and (**B**) 3 and 4 where the large points indicate the mean for each ATN category and each small data point represents a patient (A–T–N– N = 146; A–T–N+ N = 30; A–T+N– N = 4; A–T+N+ N = 5; A+T–N– N = 14; A+T–N+ N = 6; A+T+N– N = 47; A+T+N+ N = 20). (**C**) Boxplots showing the differences between the ATN categories for each PC's values where each data point represents a patient. The *p*-values have been FDR adjusted. Post hoc tests were performed with the Dunn's test with FDR *p*-value adjustment. *$p < 0.05$, **$p < 0.01$, ***$p < 0.001$, ****$p < 0.0001$. Abbreviations: ATN categories, (A) beta-amyloid, (T) phosphorylated tau, (N) neurodegeneration (+) positive or (–) negative; PCA, principal component analysis
