# Supplementary material for: Biological signatures in the Alzheimer’s continuum discriminate between diagnosis-related and -unrelated associations to ATN categories
Source: Brain Commun. 2025 Feb 21;7(2):fcaf078. doi: 10.1093/braincomms/fcaf078 (PMC11881062; doi:10.1093/braincomms/fcaf078)
Supplement: fcaf078_Supplementary_Data [file fcaf078_supplementary_data.zip › Supplementary_table_legends.docx]

**Biological signatures in the Alzheimer’s continuum discriminate between diagnosis-related and -unrelated associations to ATN categories**

Vilma Alanko^1,2^, Sára Mravinacová^3^, Anette Hall^1,4^, Göran Hagman^1,5^, Rosaleena Mohanty^1^, Eric Westman^1^, Peter Nilsson^3^, Miia Kivipelto^1,5,6,7^, Anna Månberg^3^, Anna Matton^1,2,6^

**Author affiliations:**

1. Division of Clinical Geriatrics, Department of Neurobiology, Care Sciences and Society, Karolinska Institutet, Stockholm, Sweden

2. Division of Neurogeriatrics, Department of Neurobiology, Care Sciences and Society, Karolinska Institutet, Stockholm, Sweden

3. Division of Affinity Proteomics, Department of Protein Science, KTH Royal Institute of Technology, SciLifeLab, Stockholm, Sweden

4. Institute of Clinical Medicine, University of Eastern Finland, Kuopio, Finland.

5. Theme Inflammation and Aging, Karolinska University Hospital, Stockholm, Sweden

6. Ageing Epidemiology (AGE) Research Unit, Imperial College London, London, United Kingdom

7. Institute of Public Health and Clinical Nutrition, University of Eastern Finland, Kuopio, Finland

Correspondence to: Anna Matton

Full address: Karolinska vägen 37A, QA32, 171 64 Solna, Sweden

E-mail: anna.matton@ki.se

**Running title**: Biosignatures in AD relative to diagnosis

# Supplementary table titles and legends

**Supplementary Table 1 List of antibodies used for the suspension bead array.**

**Supplementary Table 2 Contributions (%) of each protein in all principal components.**

**Supplementary Table 3 Correlations between four principal components (PC) and clinical variables.** (**A**) Correlations in the full cohort, including age adjusted correlations. (**B**) Stratified correlations between PC 1 and PC 3 and CSF Alzheimer’s disease biomarkers.
